# European sea bass show behavioural resilience to near-future ocean acidification

**DOI:** 10.1098/rsos.160656

**Published:** 2016-11-02

**Authors:** M. Duteil, E. C. Pope, A. Pérez-Escudero, G. G. de Polavieja, I. Fürtbauer, M. R. Brown, A. J. King

**Affiliations:** 1 Department of Biosciences, College of Science, Swansea University, Singleton Park SA2 8PP, UK; 2 College of Engineering, Swansea University, Bay Campus, Swansea SA1 8EN, UK; 3 Instituto Cajal, Consejo Superior de Investigaciones Científicas, Madrid, Spain; 4 Department of Physics, Physics of Living Systems Group, Massachusetts Institute of Technology, Cambridge, MA, USA; 5 Champalimaud Neuroscience Programme, Champalimaud Center for the Unknown, Lisbon, Portugal

**Keywords:** *Dicentrarchus labrax*, environmental change, fisheries, motion tracking, social behaviour

## Abstract

Ocean acidification (OA)—caused by rising concentrations of carbon dioxide (CO_2_)—is thought to be a major threat to marine ecosystems and has been shown to induce behavioural alterations in fish. Here we show behavioural resilience to near-future OA in a commercially important and migratory marine finfish, the Sea bass (*Dicentrarchus labrax*). Sea bass were raised from eggs at 19°C in ambient or near-future OA (1000 µatm *p*CO_2_) conditions and *n* = 270 fish were observed 59–68 days post-hatch using automated tracking from video. Fish reared under ambient conditions, OA conditions, and fish reared in ambient conditions but tested in OA water showed statistically similar movement patterns, and reacted to their environment and interacted with each other in comparable ways. Thus our findings indicate behavioural resilience to near-future OA in juvenile sea bass. Moreover, simulated agent-based models indicate that our analysis methods are sensitive to subtle changes in fish behaviour. It is now important to determine whether the absences of any differences persist under more ecologically relevant circumstances and in contexts which have a more direct bearing on individual fitness.

## 1. Background

Rising concentrations of carbon dioxide (CO_2_) in the atmosphere over the past 200 years have led to ocean acidification (OA) that threatens marine ecosystems [1–3]. It is predicted that by the year 2100 up to half of the seawater at the ocean's surface will show partial pressures of CO_2_ exceeding 1000 micro-atmospheres (μatm), with detrimental implications for fisheries [4]. Consequently, there is a collective drive in the scientific community to better understand how future OA may impact upon marine fisheries and ecosystems [5].

Generally, the first response of organisms to any environmental change is to modify their behaviour which, in turn, shapes ecological effects [6]. However, it is challenging to incorporate behaviour into climate change studies, and this is likely limiting our ability to forecast the impacts of change [7–9]. Given the ecological and economic ramifications of positive or negative effects on commercially important marine fisheries, it is critical to investigate the behavioural effects of near-future oceanic conditions, using robust and relevant experiments to inform management strategies [6,7]. Recent work studying a variety of marine fish species has found both negative or neutral effects of near-future OA conditions on fish behaviour [10–15]. Such contradictory results may, at least in part, be explained by the different ways experiments are designed and conducted. For instance, some researchers expose fish to OA conditions and observe fish behaviour in ambient conditions (e.g. [10,16]) while others expose fish to OA conditions and also observe fish behaviour in OA water (e.g. [11]). Overall, this makes it difficult to disentangle OA effects from any behavioural alterations that are a response to *any* changed environment.

Here, we investigate the behaviour of juvenile European sea bass, *Dicentrarchus labrax*, a commercially important fish species with global landings and total aquaculture production worth over $US 1 billion in 2012 (8990 and 153 182 tonnes from capture fisheries and aquaculture, respectively [17]). We assessed fish behaviour under three different conditions: (i) raised and tested under ambient conditions, (ii) raised and tested under near-future OA conditions, and (iii) raised under ambient conditions but tested under near-future OA conditions. We used automated video tracking [18] and calculated behavioural measures that are commonly used as measures of inter-individual differences in behaviour within populations [19,20] and are likely to be of biological and ecological relevance for obligate schooling migratory fish species like sea bass [21,22]. Significant differences in fish behaviour across our experimental conditions would provide evidence for behavioural alterations in the sea bass in response to OA [10–13,16,23]. By contrast, if fish showed statistically similar movements and interacted with their environment and each other in comparable ways, this would indicate behavioural resilience to OA. Given that the laboratory environment could constrain fish behaviour (e.g. the restriction of movement to the test arena), we also built an artificial agent-based model of the experiment, which we compared with our experimental data, allowing us to gauge whether our analysis method is sensitive to subtle changes in fish behaviour.

## 2. Material and methods

### 2.1. Subjects

Fertilized *D. labrax* eggs from a mixed spawn (multiple males and females) were incubated in water at 19°C and 585 µatm *p*CO_2_ (ambient) or 1000 µatm *p*CO_2_ (for full details of rearing conditions, see the electronic supplementary material and Pope *et al.* [24]); *n* = 180 fish from 585 µatm *p*CO_2_ conditions (mean length ± s.d. = 32.34 ± 3.12 mm), and *n* = 90 fish (mean length ± s.d. = 35.35 ± 4.61 mm) from 1000 µatm *p*CO_2_ conditions were collected from their home tanks and put in groups of *n* = 10 using a sweep net (one movement) and studied over a 10-day period from day 59 to day 68 of development, which coincides with metamorphosis into the early juvenile stage when schooling behaviour begins to develop. Following completion of experiments, fish were sacrificed by an overdose of anaesthetic (MS222; Acros Organics) and total length and weight were measured.

### 2.2. Experimental set-up

**Figure 1.**
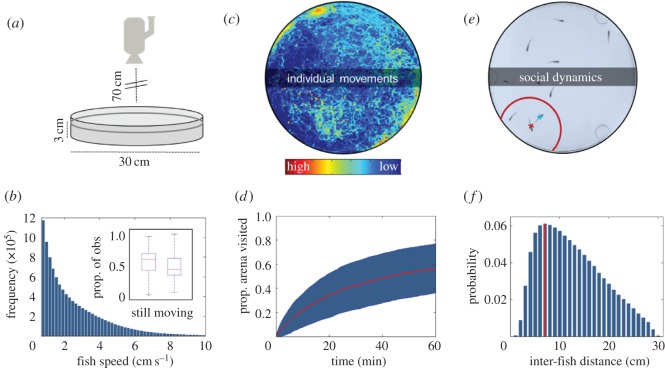
Data collection and processing. (*a*) Fish were filmed in circular test arenas placed in a larger tank with flow-through water system (see ‘Experimental set-up’ in Material and methods for details). (*b*) Frequency histogram of fish speeds (cm s^−1^) across all experiments when detected as moving; inset shows the proportion of total observations in which fish are detected as still or moving. (*c*) Example of a single fish's movement trajectory over a 60 min trial, with intensity of pixels indicating the amount of time the fish centroid spent on the pixel. (*d*) Data showing mean (red line) plus standard deviation (blue area) in exploration of the test arena (pixels explored) as a function of time for *n* = 260 fish over 26 one-hour trials. (*e*) A single frame screenshot showing the camera view of *n* = 10 fish from video collected at 25 Hz, with a focal fish highlighted by a red cross. Conspecifics within the median inter-fish distance are within the red line (*n* = 2), and the focal fish's nearest neighbour indicated by a blue cross. (*f*) Frequency histogram showing inter-fish distances for *n* = 260 fish over 26 one-hour trials; the modal inter-fish distance is indicated by the red vertical line.

Groups of *n* = 10 fish were placed in one of three circular Plexiglas ‘test arenas’ (30 cm diameter, 3 cm height) with permeable mesh bottoms (figure 1*a*). The test arenas were placed in a larger tank with a flow-through water system to ensure conditions were maintained. Test arenas were separated with opaque plastic partitions and surrounded by a custom-built aluminium frame and white screen (PhotoSEL BK13CW White Screen). A Panasonic HDC-SD60 HD video camera (Panasonic Corporation of North America, Secaucus, NJ, USA) was positioned above each test arena (figure 1*a*). Four photographer's lights (each with 4 × 25 W 240 V 6400 K True Day light bulbs) lit the arenas from the outside, hence dispersing light evenly over the arenas and enabling optimum conditions for video recording. Video recordings were started approximately 60 s after the fish were placed into the arena and arenas were recorded for 60 min of footage (90 000 frames at 25 Hz; representing 90 000 data points per fish) which was subsequently tracked to provide *x*, *y* coordinates (below). We tested groups of fish reared at near-future OA conditions (‘OA fish’, *n* = 9 groups, *n* = 90 fish), ambient ocean conditions (‘ambient fish’, *n* = 9 groups, *n* = 90 fish) and fish reared under ambient conditions but tested in OA water (‘ambient fish tested in OA’, *n* = 8 groups, *n* = 80 fish, because of a processing error of one video file).

### 2.3. Generating *x*, *y* fish coordinates

Experiments were recorded as AVCHD videos, and converted into mp4 using Freemake (v. 4.1.5.4) to allow tracking of fish position using idTracker [18]. idTracker software extracts a characteristic fingerprint from each individual being filmed, and then uses these fingerprints to identify every individual throughout the video. For each video, we restricted the analysis to the arena, and selected the minimum size and intensity threshold in a way that we detected the 10 fish and no artefacts in ten frames randomly chosen in the video. After tracking, idTracker returns the *x, y* coordinates for each individual fish at temporal resolution of 25 Hz (i.e. the frame rate). The limited water depth (3 cm; figure 1*a*) ensures accuracy in our tracking in two dimensions and calculation of subsequent behavioural variables (see below), but also restricted fish movement in the vertical plane.

### 2.4. Processing and interpolation of *x*, *y* fish coordinates

Some errors were detected in the *x, y* coordinates generated by idTracker and so we undertook the following data-processing stages in Matlab (v. R2014b): (i) scaling: we normalized the images so that all the data we processed were at a scale of 0.03 cm per pixel, with the centre at (0;0) and diameter of 1000 pixels (29 cm). This enabled us to delete any points that lay outside the arena which typically occurred when the fish swam near the edge of the arena and their reflection was detected by idTracker as another fish. (ii) Fish label swapping: occasionally a fish was confused with another fish or with an artefact by idTracker.

For each frame and for each tracked object (fish), we calculated the instantaneous speed *s_t_* ($s_{t}=\sqrt{{\left(x_{t}-x_{t-1}\right)}^{2}+{\left(y_{t}-y_{t-1}\right)}^{2}}$, with *x* and *y* being the coordinates of the fish and *t* designating the current time frame). We compared this speed with a maximum possible speed, considered to be the fastest speed detected during manual tracking of videos, at 0.91 m s^−1^. This speed is coherent with other observations of maximum speed for juvenile sea bass at this temperature [25]. These two stages of post-processing eliminated 0.1% of data points that were considered as erroneous. Third, we interpolated the sections of missing points in our *x, y* coordinates that were sufficiently short (see electronic supplementary material, figure S1, for an example, for processing for one fish in one experiment). For every sequence of missing data shorter than one second and for which we knew the position of the fish for the five frames just before and just after that sequence, we computed a piecewise cubic interpolation to determine the polynomial which best approximated the missing values. This last step allowed us to determine 104 143 more data points, giving us a total of 2 563 633 frames with all fish, which represented 76% of the entire dataset (31 144 046 data points; 8 937 683 for the ambient condition, 8 689 475 for the OA condition, and 13 516 888 for the ambient tested in OA condition).

### 2.5. Behavioural variables

We extracted data on fish activity (median speed and proportion of time spent still), how fish reacted to the test arena (proportion of time near the edge of the arena, time taken to explore 10% of the arena, and overall percentage of the arena explored), and associations of fish with each other (median distance to nearest neighbour, and mean number of local neighbours) (see figure 1*b*–*e* for an overview of these variables).

Fish speed at each frame $\left(s_{t}\right)$ was calculated as described above. For our analyses, we calculated the median *s_t_* across all frames and fish. We considered that a fish stayed still between two frames if its movement was less than a pixel, which amounts to a speed of 0.7 cm s^−1^, and used this to calculate the proportion of time spent still during experiments. The proportion of time fish spent near the arena edge was based on the proportion of time fish were within 3 cm (length of fish to nearest cm) of the arena edge (20% of the radius of the arena). To determine how much of the arena fish explored within 10 min, and over the whole experiment, we partitioned each test arena into a grid of 1 × 1 cm squares (total number of squares per arena = 788; electronic supplementary material, figure S1); 100% exploration meant that the fish had entered each of the 788 squares at least once. To describe how fish interacted with each other, we calculated the distance of each fish to all other fish in every frame, and also computed the distance to the closest neighbour. Using these data, we determined the median distance between all fish for each trial. Over the entire dataset median distance was 6.9 cm and, for each fish in each frame, we then calculated the number of fish within 6.9 cm of a focal fish. For analyses, we used the mean proportion of the group that was within a focal fish's radius (less than 6.9 cm).

### 2.6. Simulation model

Because the simple environment in which the fish were tested could have constrained fish behaviour in some way (e.g. due to the geometry of the environment), we built an artificial model of our experiment. Like in the real experiment, we placed *n* = 10 fish in an arena equivalent to that used in our experiments (figure 1) and determined the position of every fish in consecutive time intervals (frames) as: x(t+1)=x(t)+v(t)×cos⁡(θ(t)+ω(t)) and y(t+1)=y(t)+v(t)×sin⁡(θ(t)+ω(t)), where *x* and *y* are the coordinates of the fish, *θ* is the current orientation of the fish, and *v* and *ω* are, respectively, speeds and angular speeds uniformly drawn at random from the distributions of speeds and angular speeds observed for the real fish. This effectively builds the simplest model of fish movement. We ran this model for 60 min, for the same number of replicates (*n* = 26) as in our experiment to produce a dataset of equivalent size to our experimental data and computed the same variables we measured with the real fish, except fish *median speed* and *time spent still*, which were directly coded in our model and, therefore, on average, identical (electronic supplementary material, figure S2). Because any speed and angular speed can be selected (from those observed in real fish), it was possible that the computed trajectory of the simulated fish fell outside the test arena. In this case, these coordinates were replaced with the symmetrical point along the edge of the arena.

### 2.7. Data analysis

Because our seven fish variables (see above) were correlated, we normalized variables and combined them into principal component scores with orthogonal rotation (varimax) using principal components (PCs) analysis [26]. To test for differences in the behaviour of fish across our three experimental conditions, we fitted three linear mixed models (LMMs) with PC1 (model 1), PC2 (model 2) and PC3 (model 3) as the response variable, and experimental condition (‘ambient’, ‘OA’, ‘ambient tested in OA’) as a categorical fixed effect. We fitted experiment number as a random effect to control for the potential non-independence of data because we observed three experimental arenas at a time.

To test for differences in the behaviour of our real fish compared with our simulated fish, we ran five linear models, one for each of the variables that were not used to generate the simulation dataset: (i) proportion of time near the arena edge, (ii) time taken to explore 10% of the arena, (iii) overall % of the arena explored*,* (iv) median distance to nearest neighbour, and (v) mean number of local neighbours, and fitted data type (real, simulation) as a fixed effect.

All statistical analyses were conducted in the R environment [27] and LMMs fitted using the package lme4 (linear modelling) [28]. The level of significance was set at *α *< 0.05 and model diagnostics were performed using graphical procedures (Q–Q plot and standardized residuals versus fitted values).

## 3. Results

**Figure 2.**
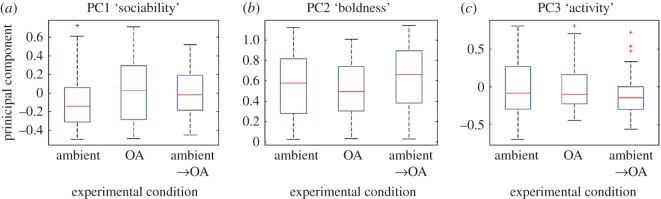
Behavioural resilience to near-future ocean acidification. Data variables extracted from tracked fish trajectories (figure 1*c*) were normalized and combined into principal components. Linear mixed models revealed that treatment type does not predict variation in (*a*) PC1 ‘sociability’, (*b*) PC2 ‘boldness’ or (*c*) PC3 ‘activity’. See table 1 for details of how behavioural variables load onto PC axes and table 2 for statistical comparisons.

Our seven correlated behavioural variables produced three uncorrelated PCs that represented 84.7% of the variance in our data (table 1). Fish that scored high on PC1 tended to (i) be close to other fish, (ii) have a high number of local neighbours, and (iii) explore less of the arena. Fish that scored high on PC2 tended to spend most of their time at the edge of the arena. Fish that scored high on PC3 tended to spend little time still, and explored a large proportion of the arena. On this basis we labelled PC1 ‘sociability’, PC2 ‘boldness’, and PC3 ‘activity’. All three PCs were statistically similar across the three treatments (*p* > 0.05; figure 2; table 2).

**Table 1.** Factorial decomposition of the first three principal components (PCs). These three PCs represent 84.7% of the variance in the data. The most important variables for each component are in italics.

variable	PC1 ‘sociability’	PC2 ‘boldness’	PC3 ‘activity’
median speed	0.025	−0.031	*0*.*520*
proportion of time spent still	−0.026	0.001	*−0*.*709*
proportion of time near the arena edge	0.064	*0*.*984*	−0.021
time taken to explore 10% of the arena	0.245	0.098	−0.114
overall % of the arena explored	*−0*.*604*	0.134	0.361
median distance to nearest neighbour	−0.271	−0.049	−0.137
mean number of local neighbours	*0*.*704*	−0.026	0.254

**Table 2.** Results of model testing for differences in the behaviour of fish across our experimental conditions. Presented are the estimates, associated standard error (s.e.), *t*-value and *p*-value with respect to PC1 ‘sociability’, PC2 ‘boldness’ and PC3 ‘activity’.

variable	estimate	s.e.	*t*-value	*p*-value
PC1				
intercept	−0.096	0.107		
ambient versus OA	0.144	0.144	1.002	0.316
ambient versus ambient → OA	0.081	0.106	0.762	0.446
PC2				
intercept	0.583	0.047		
ambient versus OA	−0.079	0.063	−1.254	0.210
ambient versus ambient → OA	0.040	0.045	0.882	0.377
PC3				
intercept	−0.026	0.055		
ambient versus OA	0.003	0.078	0.040	0.968
ambient versus ambient → OA	−0.093	0.081	−1.147	0.252

**Figure 3.**
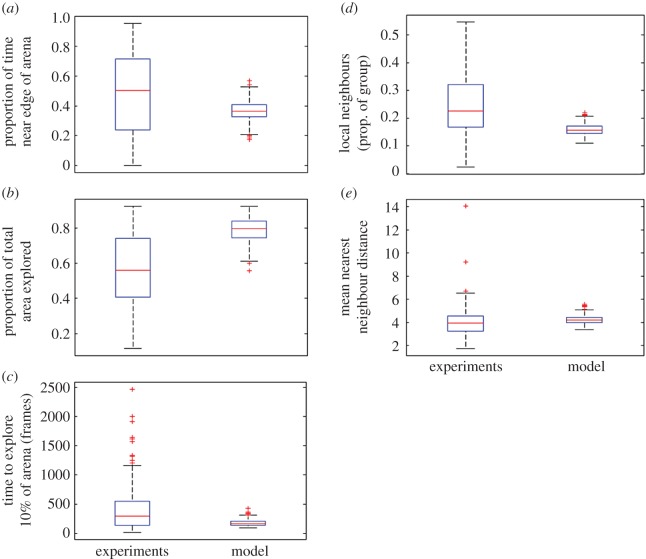
Differences in behaviour, reaction to the environment and interspecific interactions for sea bass (experiments) and simulated fish (agent-based model). Experimental data with real fish suggest that they (*a*) are more likely to be found close to the edge of the arena, (*b*) explore less of the arena, (*c*) explore more quickly, (*d*) have higher number of neighbours nearby and (*e*) are closer to other fish. Statistical comparisons are presented in table 3.

Comparing our experimental data with our simulated agent-based model, we found statistically significant differences for all variables tested (*p* < 0.05; table 3). Compared with our model fish, real fish showed a greater tendency to follow the edge of the arena (figure 3*a*; table 3), explored a smaller proportion of the arena (figure 3*b,c*; table 3) and showed a greater attraction to their neighbours, resulting in greater cohesion within groups (figure 3*d*; table 3) and shorter distances to nearby neighbours (figure 3*e*; table 3). The real fish also showed greater variability in these measures than the model fish (Levene's test: *p* < 0.001 in all cases). An example movie of the real fish data and simulated fish data is provided as a movie in the electronic supplementary material.

**Table 3.** Results of model testing for differences in the behaviour of real and simulated (model) fish for different behavioural variables. Presented are the estimates, associated standard error (s.e.), *t*-value and *p*-value.

variable	estimate	s.e.	*t*-value	*p*-value
*proportion of time near the arena edge*				
real versus simulated data	0.123	0.018	6.894	<0.001
*time taken to explore 10% of the arena*				
real versus simulated data	230.7	23.68	9.745	<0.001
*overall % of the arena explored*				
real versus simulated data	−0.223	0.013	−16.65	<0.001
*proportion of local neighbours*				
real versus simulated data	0.090	0.007	13.56	<0.001
*distance to nearest neighbour*				
real versus simulated data	−0.258	0.076	−3.380	<0.001

## 4. Discussion

We investigated the effects of near-future oceanic conditions upon behaviour in a commercially important and migratory marine finfish, the European sea bass. Fish showed statistically similar movements, interaction with their environment, and interaction with each other, regardless of whether they were raised and tested in ambient conditions, OA conditions, or raised under ambient conditions and tested in OA water. Overall, this indicates behavioural resilience to OA, supporting work on individual subjects showing that increased *p*CO_2_ similarly has no significant effect on larval morphology in this species [24], and work on individual subjects (rather than groups) investigating swimming activity and/or kinematics in juvenile or larval fish; for example, studies of cobia (*Rachycentron canadum*) [29], mahi-mahi (*Coryphaena hippurus*) [30], Atlantic cod (*Gadus morhua*) [31] and herring (*Clupea harengus* L.) [32] do not find an effect of elevated CO_2_. For an obligate schooling migratory fish like sea bass, our finding is good news, and suggests that we can be optimistic about their ability to cope with anthropogenic changes to their environment in the near future.

Since the simple environment in which the fish were tested in the laboratory could somehow constrain behaviour, we also built an agent-based model of the experiment which we compared with our experimental data. Comparing the model and real data allowed us to gauge whether our analysis method is sensitive to subtle changes in fish behaviour. Our results strongly suggest that the juvenile sea bass are interacting with one another and the environment in biologically relevant ways, and that these behaviours result in very different behavioural patterns that emerge from a simple model of fish that move with the same speed and turning angles.

Rearing fish in a competitor- and predator-free industrial setting where fish are not resource limited may have restricted our ability to detect differences. In fact, the importance of risk-taking behaviours (PC2) and social tendencies (PC1) measured here will likely be exaggerated during migratory movements [33–35] or under predation pressure [20,36]. Therefore, it will be important to determine whether the absences of any differences that we find here persist in adult fish and under more ecologically relevant circumstances and in contexts which have a more direct bearing on individual fitness. Indeed, while individual variation in behavioural responses to high CO_2_ clearly exists (e.g. [37]), it is unknown how quickly favourable genotypes enabling the expression of particular behaviours could be established in populations. Extending the sorts of experiments presented here to explore within- and between-individual variation in behaviour across time and contexts (and preferably across generations) would enable researchers to address such questions. Similarly, our experiments were specifically designed to investigate the effects of OA conditions on sea bass behaviour as if they were born into and live under such conditions in the near future. Of course, exposing the same fish to an acute change in OA may well have resulted in a behavioural change for the variables we investigated, or in other behaviours. For example, temperate wrasses (*Ctenolabrus rupestris*) exposed to OA (but tested in control water) show reduced avoidance of predator odour but no change in behavioural lateralization or swimming activity compared to control fish [38], and juvenile Atlantic cod (*Gadus morhua*) avoid OA water irrespective of how long they experience elevated CO_2_ [31]. Exposing subjects to acute shifts in *p*CO_2_ provides interesting case studies which add to our understanding of behavioural responses to rapid environmental change, but their ecological relevance to real-world OA is not always clear.

Overall, while we urge caution in over-generalizing our findings, our experimental investigations of the effects of near-future oceanic conditions on juvenile sea bass behaviour represent the most comprehensive study of its kind to date. We have used sophisticated automated tracking from video to produce a large and robust dataset to compare fish under different experimental conditions. We hope that the methodology and approach we have adopted here will inspire the development of even more elaborated experiments and models to understand how future OA may impact upon individual and social behaviour and how behaviour may shape ecological effects.

## Supplementary Material

Electronic Supplementary Material: Details of fish rearing conditions and three Supplementary Figures (Figures S1-S3) are provided

## Data accessibility

Data are available from the Dryad Digital Repository http://dx.doi.org/10.5061/dryad.2dc8k [39].
