# Supplementary material for: European sea bass show behavioural resilience to near-future ocean acidification
Source: R Soc Open Sci. 2016 Nov 2;3(11):160656. doi: 10.1098/rsos.160656 (PMC5180154; doi:10.1098/rsos.160656)
Supplement: Electronic Supplementary Material: Details of fish rearing conditions and three Supplementary Figures (Figures S1-S3) are provided [file rsos160656supp1.docx]

**European sea bass show behavioural resilience to near-future ocean acidification**

**ELECTORNIC SUPPLEMENTARY MATERIAL**

M. Duteil, E.C. Pope, A. Pérez-Escudero, G.G. de Polavieja,

I. Fürtbauer, M.R. Brown, A.J. King

**Supplementary Methods - Rearing Conditions**

Two seawater systems were connected to a 60 000 L recirculating aquaculture system. Each system comprised a 1500 L header tank feeding into 3 x 150 L experimental tanks. Fertilised *Dicentrarchus labrax* eggs from a mixed spawn (multiple males and females) purchased from Écloserie Marine de Gravelines, France, were placed in 6 x 10 L incubators, each held within one of the experimental tanks at 13 °C, ambient *p*CO_2_ and under a 12 h light:12 h dark photoperiod (median light = 6.5 µmol photon m^-2^ s^-1^, range = 4.2 – 12.4 µmol photon m^-2^ s^-1^). After hatching, 1,000 larvae were transferred from each incubator into the respective experimental tank and the incubators removed. The *p*CO_2_ in one system was then gradually increased to 1,000 µatm over 24 h and the temperature in both systems increased to 19 °C at the rate of 1 °C d^-1^. Elevated *p*CO_2_ was attained *via* a computerised feedback system which monitored seawater pH_NBS_ in the header tank and regulated the injection of compressed CO_2_ gas. Fish were maintained for an experimental period of 75 d, fed *ad libitum* on rotifers (*Brachionus plicatilis*) between days 2 and 26 and enriched brine shrimp (*Artemia salina*) from day 9 twice daily. Larvae were sampled at regular intervals for other studies. Salinity in the experimental tanks was 28.17 ± 0.22 (mean ± 1SD) measured according to the practical salinity scale and temperature was 18.83 ± 0.49 °C (mean ± 1SD) during the incubations.

Temperature and pH_NBS_ were measured daily in each experimental tank using a WTW type pH/Cond 340i probe, calibrated daily using a NIST/DIN-traceable calibration (WTW technical buffers at 7.0 and 10.0). Total alkalinity (TA), was also measured in each header tank but less frequently (typically twice a week) using open-cell potentiometric titration (Total Alkalinity AS-ALK2 Gran Titration System, Apollo SciTech Inc.) with a high precision pH meter (Orion 3-star, Thermo Scientific) and ROSS combination pH glass electrode (Orion 8102BNUWP, Thermo Scientific). Full details of the calibration procedure for the titration system are available from Pope et al. (2014) cited in the main text. Silicate and phosphate concentrations were also measured using a segmented-ﬂow injection autoanalyzer (Bran Luebbe, SEAL Analytical Ltd, Fareham, Hampshire, UK). *p*CO_2_ in the 1,000 µatm *p*CO_2_ system was 1,030 ± 89 µatm (mean ± 1SD) during the incubation, whilst the ambient system maintained a *p*CO_2_ of 585 ± 50 µatm (mean ± 1SD).


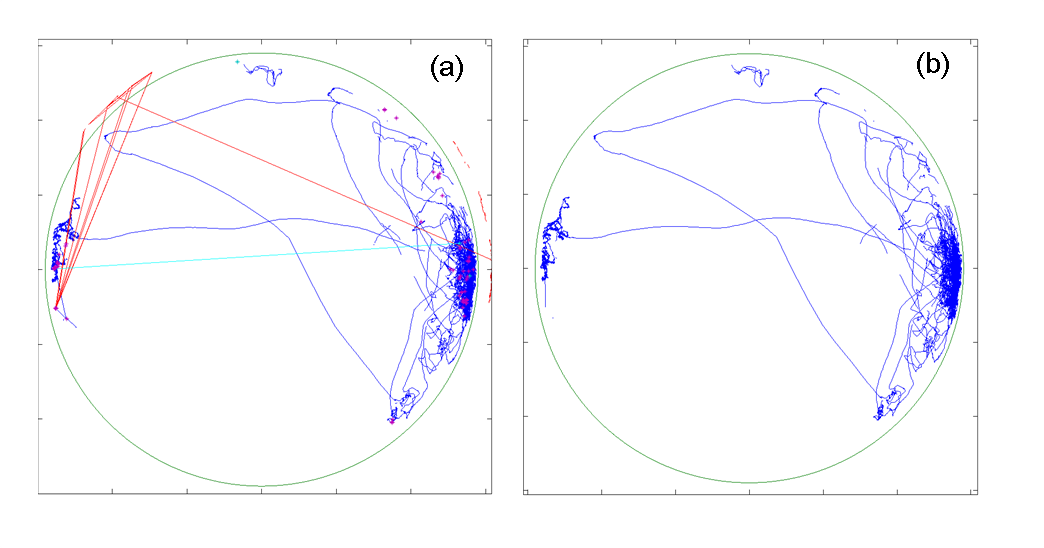


**Figure S1. Data-processing and error elimination.** Example of erroneous data removal for a series of n=90,000 data points (i.e. one fish in one trial). Points are removed because they lie outside the test arena (n=510; red); because speed is excessive (n=8; cyan); or because they are isolated points that are not associated with the fish trajectory (n=102; purple). The remaining points (in blue) are considered correct. (b) Trajectory of the fish after processing.

**
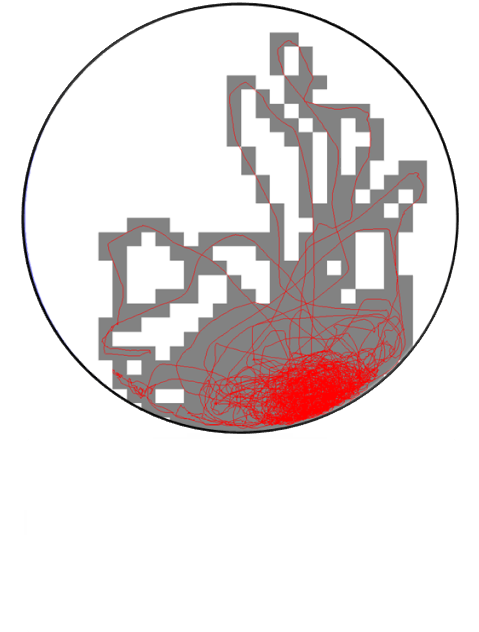
**

**Figure S2. Example of a single fish’s trajectory and the corresponding explored area.** Fish trajectory is shown in red, the white area represents the surface that is considered to be inside the tank, and the grey area represents the fraction of this area which has been observed by the fish.

***
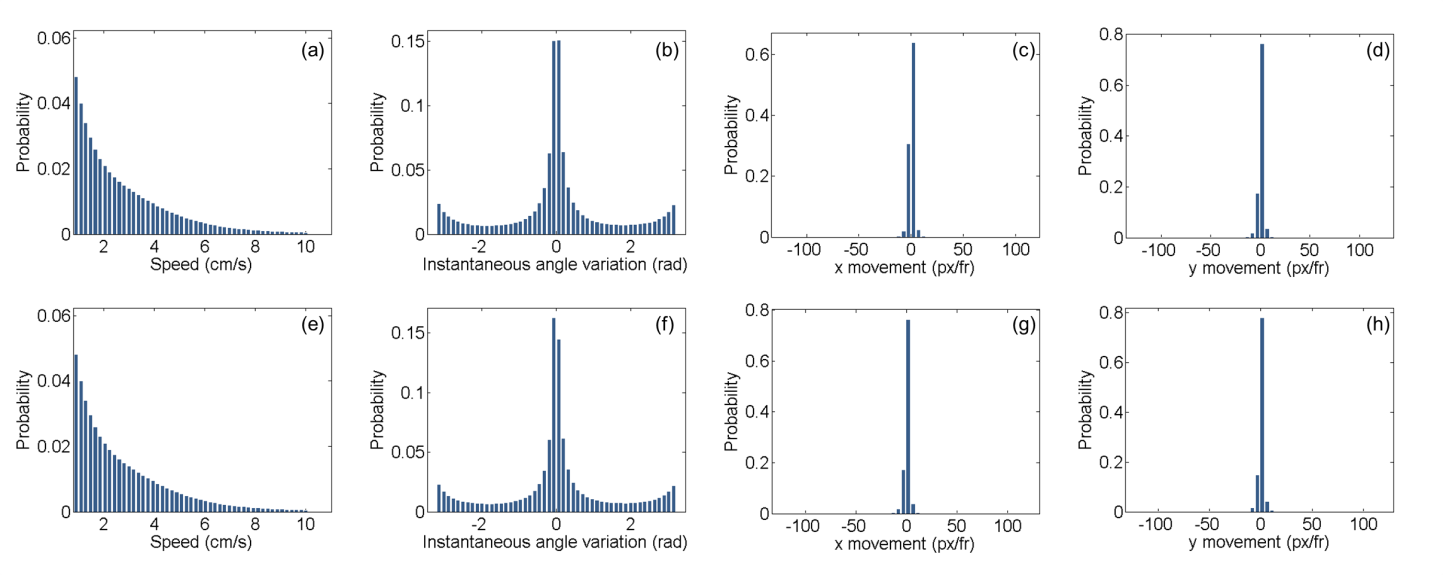
***

**Figure S3 Comparison of speed and movement data from real fish and simulated (model) fish.** (a)-(d) are data from the real fish, and (e)-(h) are data from our simulated fish. Information for the entire datasets are presented.
